# Origin, Evolution, and Diversification of the Expansin Family in Plants

**DOI:** 10.3390/ijms252111814

**Published:** 2024-11-03

**Authors:** Zhizhan Wang, Jinbiao Cao, Nan Lin, Jiaming Li, Yazhou Wang, Weibin Liu, Wen Yao, Yang Li

**Affiliations:** College of Life Sciences, Henan Agricultural University, Zhengzhou 450046, China

**Keywords:** expansin, cell wall, origin, evolution, diversification

## Abstract

The cell wall is a crucial feature that allows ancestral streptophyte green algae to colonize land. Expansin, an extracellular protein that mediates cell wall loosening in a pH-dependent manner, could be a powerful tool for studying cell wall evolution. However, the evolutionary trajectory of the expansin family remains largely unknown. Here, we conducted a comprehensive identification of 2461 expansins across 64 sequenced species, ranging from aquatic algae to terrestrial plants. Expansins originated in chlorophyte algae and may have conferred the ability to loosen cell walls. The four expansin subfamilies originated independently: *α*-expansin appeared first, followed by *β*-expansin, and then expansin-like A and expansin-like B, reflecting the evolutionary complexity of plant expansins. Whole genome duplication/segmental duplication and tandem duplication events greatly contributed to expanding the expansin family. Despite notable changes in sequence characteristics, the intron distribution pattern remained relatively conserved among different subfamilies. Phylogenetic analysis divided all the expansins into five clades, with genes from the same subfamily tending to cluster together. Transcriptome data from 16 species across ten lineages and qRT-PCR analysis revealed varying expression patterns of *expansin* genes, suggesting functional conservation and diversification during evolution. This study enhances our understanding of the evolutionary conservation and dynamics of the expansin family in plants, providing insight into their roles as cell wall-loosening factors.

## 1. Introduction

Green plants (Viridiplantae) are widely distributed in aquatic and terrestrial ecosystems. During the colonization of land, ancestral green plants evolved their cell walls to adapt to terrestrial environments. The plant cell wall is an elaborate structure that surrounds the plasma membrane, providing structural support and protection to the plant cell. Major components of the cell wall include cellulose, hemicellulose, and pectin, which are organized into primary and secondary cell walls. The primary cell wall, which controls cell expansion and elongation, has a thickness ranging from approximately 0.1 to 10 μm [1]. Following the cessation of cell growth, a robust secondary cell wall begins to deposit inside the primary cell wall, providing additional protection, rigidity, and strength. Currently, many cell wall-related genes have been documented. In plants, cellulose, composed of *β*-1,4 glucans, is an important biopolymer in the cell wall. Plasma membrane-embedded cellulose synthases A (CesAs) directly catalyze the elongation of the *β*-1,4 glucan chains and align them into protofibrils [2]. The cryo-electron microscopy (cryo-EM) structures of PttCesA8 and GhCesA7 have been elucidated to enhance our understanding of the molecular mechanism underlying plant cellulose synthesis [3,4]. Xyloglucan is the most abundant hemicellulose in the primary wall of most seed plants [5]. Cellulose synthase like-C (CSLC) in *Arabidopsis thaliana*, which is involved in synthesizing the xyloglucan backbone, has been identified [6]. Homogalacturonan (HG), a main type of pectin in the primary cell wall, is methyl-esterified in the Golgi and demethyl-esterified after secretion into the cell wall [7]. Pectin methylesterase (PME) and pectin methylesterase inhibitor (PMEI) regulate the demethylesterification of HG in two ways: non-blockwise and blockwise [8].

In addition to the three major cell wall components (cellulose, hemicellulose, and pectin), there are cell wall-related proteins, such as expansin, wall-associated kinase, and pectate lyase, that play numerous important roles in cell wall dynamics [9,10,11]. Expansin, first identified in cucumber hypocotyls, is a classic and important cell wall protein that facilitates cell wall loosening without causing the lysis of wall polymers [12,13]. Research has shown that ZmEXPB1 specifically binds to glucuronoarabinoxylan to loosen the cell wall in maize [14]. Typical expansin proteins possess two conserved functional domains: the DPBB domain (N-terminal domain, 120–135 amino acids), which is similar to family-45 glycosyl hydrolase (GH45); and the CBM63 domain (C-terminal domain, 90–120 amino acids), which is homologous to the family-63 carbohydrate-binding module (CBM63) [13,15]. Both domains are preceded by a signal peptide consisting of 20–30 amino acids. Based on phylogenetic analysis and sequence characteristics, the expansin family is divided into four subfamilies: *α*-expansin (EXPA), *β*-expansin (EXPB), expansin-like A (EXLA), and expansin-like B (EXLB). To date, EXPA and EXPB have been associated with cell enlargement, while EXLA and EXLB have received comparatively less attention [16,17].

In plants, immunolocalization studies have demonstrated that expansins are mainly localized to the cell wall [18,19]. Expansins are highly expressed in developing tissues and organs where cell wall loosening or disassembly occurs, such as in growing roots, expanding leaves, and developing seeds, fruits, or fibers [20,21,22,23,24]. In many plant species, the biological functions of expansins have been confirmed, including their roles in regulating root growth, leaf/pollen tube development, grain size, fruit ripening, and stress tolerance. During lateral root development in *Arabidopsis*, the auxin-responsive gene *AtEXPA1* affects the division and expansion of pericycle cells [22]. In wheat, the *EXPA* gene *TaExpA6*, which is normally expressed in roots, was ectopically expressed in early developing seeds, resulting in larger grains without affecting the grain number [21]. Tomato *Exp1* and endoglucanase *Cel2* work together to enhance fruit softening and facilitate cell wall disassembly [23]. Additionally, expansins play a key role in responding to abiotic and biotic stresses in crops such as rice, wheat, maize, soybean, peanut, and cotton [17,25,26,27,28,29].

Advances in sequencing techniques for plant genomics over the past decade have been impressive, enabling the elucidation of the evolution and phylogeny of some pivotal cell wall-related genes in plants, such as cellulose synthase, PME/PMEI, and glucuronic acid substitution of xylan (GUX) [30,31,32]. However, a comprehensive understanding of the evolution of the plant expansin family at the genomic level is still lacking. In this study, we investigated the presence and absence of expansins by mining the genomes of 64 species, ranging from aquatic algae to land plants. Expansins were found in Chlorophyta and Streptophyta but are missing in all the red algae studied. Furthermore, we explored the expansion mechanism, sequence characteristics, and intron distribution pattern of expansins, as well as their evolutionary conservation. Finally, we focused on the functional conservation and diversification of the expansin family during evolution using transcriptome data from 16 representative species and conducting qRT-PCR analysis.

## 2. Results

### 2.1. The Origination of Expansin Genes in Plants

**Figure 1:**
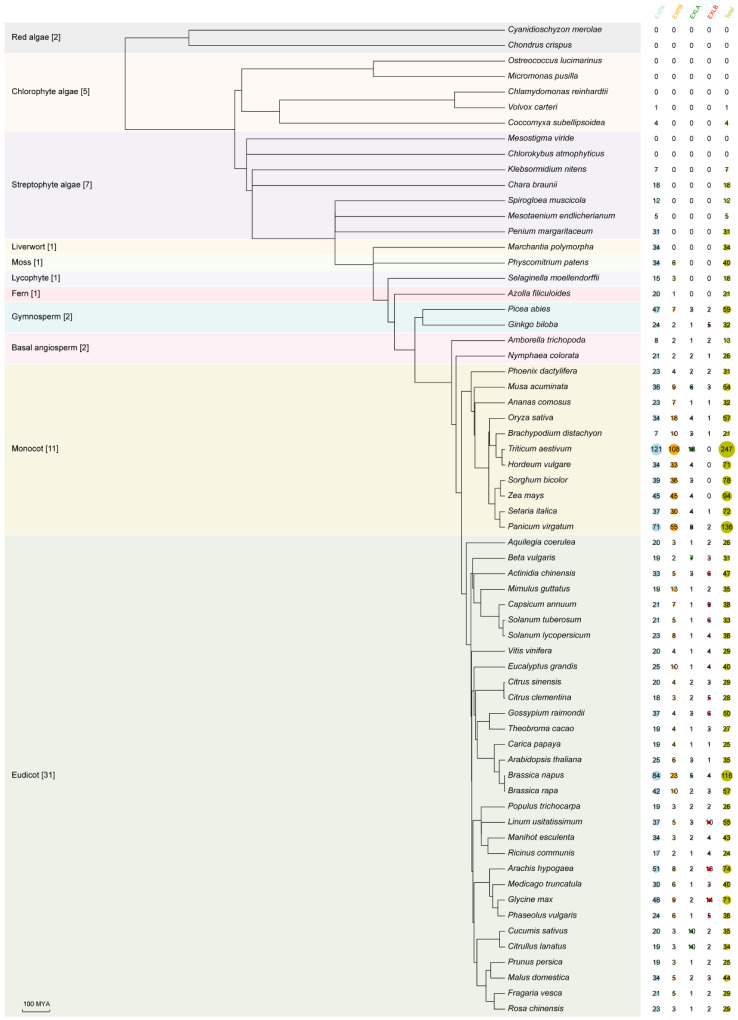
Occurrence of the expansin family among green plants. The number of *expansin* genes for each species is shown. The four previously defined expansin subfamilies (EXPA, EXPB, EXLA, and EXLB) are indicated. The species tree was constructed using TimeTree 5 (http://www.timetree.org/) [34]. The scale bar represents divergence time (million years ago, MYA).

To better investigate the origin of *expansin* genes in plants, we selected 64 representative species, including red algae (Rhodophyta), green algae (Chlorophyta), streptophyte algae (Charophyta), and land plants (Embryophyta) (Table S1). The rhodophytes included two species, the chlorophytes included five species, the streptophyte algae included seven species, and the land plants included 50 species classified into eight lineages (i.e., liverworts, mosses, lycophytes, ferns, gymnosperms, basal angiosperms, monocots, and eudicots) [33]. A total of 2461 expansin proteins belonging to four subfamilies were identified utilizing BLAST, HMMER 3.3.1, and NCBI CDD (Table S2). Of the 64 species examined, the number of expansin proteins varied significantly, ranging from 0 to 247 (Figure 1). No expansin proteins were detected in two rhodophytes (*Cyanidioschyzon merolae* and *Chondrus crispus*), three chlorophytes (*Ostreococcus lucimarinus*, *Micromonas pusilla* and *Chlamydomonas reinhardtii*), and two streptophyte algae (*Mesostigma viride* and *Chlorokybus atmophyticus*). To gain additional insights into the origin of *expansin* genes, the genomes of the seven species were analyzed. We identified 17 protein sequences encoding the DPBB domain with significant e-values (<1 × 10^−5^) in the genomes of four species (*C*. *crispus*, *O*. *lucimarinus*, *M*. *pusilla* and *C*. *reinhardtii*), whereas the CBM63 domain was not detected (Figure S1, Table S3). Surprisingly, the rhodophyte *C. merolae* and the two streptophyte algae, *M. viride* and *C. atmophyticus*, were found to lack both the DPBB and CBM63 domains (Figure S1). Subsequently, one expansin protein was identified in the green algae *Volvox carteri* and four in *Coccomyxa subellipsoidea*. In the land plants, expansin proteins were found to be ubiquitously present, suggesting that expansin proteins first appeared in chlorophytes during the evolutionary process.

The published literature shows that expansin proteins are grouped into four subfamilies (EXPA, EXPB, EXLA, and EXLB) based on sequence characteristics [13,15]. We next investigated the origin of the four subfamilies (Figure 1). EXPA was first identified in the chlorophytes (*V*. *carteri* and *C*. *subellipsoidea*) and appeared widely in the streptophyte algae and land plants. EXPB was first present in the moss (*Physcomitrium patens*), comprising six members. EXLA and EXLB were absent in the chlorophytes, streptophyte algae, liverworts, mosses, lycophytes, and ferns, but were first found in the gymnosperms (*Picea abies* and *Ginkgo biloba*). All four subfamilies were present in the seed plants (gymnosperms, basal angiosperms, monocots, and eudicots). These data suggest that the emergence times of EXPA, EXPB, and EXLA/EXLB were inconsistent, with EXPA occurring earliest in chlorophytes. EXPB appeared after the divergence of liverworts and mosses, while EXLA and EXLB were limited to seed plants.

### 2.2. Evolutionary Enlargement of the Expansin Family

Gene-family expansion is a well-known contributor to the diversity of gene functional characteristics during evolution. To understand the enlargement of the expansin family in plants, we first observed the number of expansin proteins across different lineages (Table S4). The average number of expansin proteins per lineage varied significantly, ranging from 2.5 members in the chlorophytes to 14.6 in the streptophyte algae, 34 in the liverworts, 40 the in mosses, 18 in the lycophytes, 21 in the ferns, 45.5 in the gymnosperms, 19.5 in the basal angiosperms, 81.18 in the monocots, and 40.23 in the eudicots. The expansin proteins showed a trend of gradual expansion from chlorophyte algae to mosses, contrasting with the more variable expansion observed in the other six lineages of land plants during evolution.

Natural diploids and polyploids are prevalent in plants. Significant variation in the number of *expansin* genes was observed among 43 diploid species, ranging from 13 in *Amborella trichopoda* to 94 in *Zea mays*, reflecting the occurrence of gene expansion events (Figure 1). When the *expansin* numbers in three tetraploid species (*Panicum virgatum*, *Brassica napus* and *Arachis hypogaea*) and one hexaploid species (*Triticum aestivum*) were normalized to the diploid level, *T. aestivum*, *P. virgatum*, and *B. napus* still had more than 50 members each.

By investigating the number of each expansin subfamily, we found that the number of EXPA was significantly larger than those of EXPB, EXLA, and EXLB in most of the species studied. Exceptions to this trend were observed in *Brachypodium distachyon*, *T*. *aestivum*, *Hordeum vulgare*, *Sorghum bicolor*, and *Z*. *mays* (Figure 1). The largest average number of EXPB was found in Gramineae plants, with 41,875 members. Notably, EXLB was absent in four Gramineae crops (*T. aestivum*, *Hordeum vulgare*, *S. bicolor*, and *Z. mays*), which is somewhat surprising.

**Figure 2:**
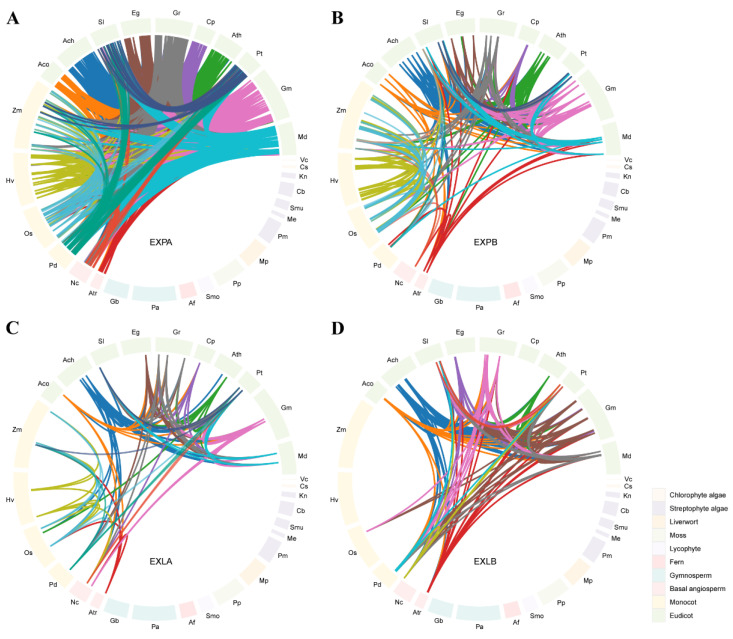
Schematic representations of the collinear relationships of *expansin* genes among 29 of the 57 species. (**A**) EXPA subfamily; (**B**) EXPB subfamily; (**C**) EXLA subfamily; (**D**) EXLB subfamily. The length of each colored bar in the circle indicates the number of *expansin* genes in a species. *Expansin* genes within each species are arranged clockwise along a colored bar, according to their genomic coordinates. Each colored line represents a colinear *expansin* gene pair. The species names are as follows: *Volvox carteri* (*Vc*), *Coccomyxa subellipsoidea* (*Cs*), *Klebsormidium nitens* (*Kn*), *Chara braunii* (*Cb*), *Spirogloea muscicola* (*Smu*), *Mesotaenium endlicherianum* (*Me*), *Penium margaritaceum* (*Pm*), *Marchantia polymorpha* (*Mp*), *Physcomitrium patens* (*Pp*), *Selaginella moellendorffii* (*Smo*), *Azolla filiculoides* (*Af*), *Picea abies* (*Pa*), *Ginkgo biloba* (*Gb*), *Amborella trichopoda* (*Atr*), *Nymphaea colorata* (*Nc*), *Phoenix dactylifera* (*Pd*), *Oryza sativa* (*Os*), *Hordeum vulgare* (*Hv*), *Zea mays* (*Zm*), *Aquilegia coerulea* (*Aco*), *Actinidia chinensis* (*Ach*), *Solanum lycopersicum* (*Sl*), *Eucalyptus grandis* (*Eg*), *Gossypium raimondii* (*Gr*), *Carica papaya* (*Cp*), *Arabidopsis thaliana* (*Ath*), *Populus trichocarpa* (*Pt*), *Glycine max* (*Gm*), and *Malus domestica* (*Md*).

Whole genome duplication (WGD), segmental duplication, and tandem duplication have been proven critical for gene family expansion during genome evolution. To trace the expansion history of the expansin family in plants, we identified 21,922 collinear blocks containing 1771 *expansin* genes through intergenomic comparison within 44 angiosperm species (two basal angiosperms, 11 monocots, and 31 eudicots) using MCScanX. It indicated that these genes were generated through WGD or segmental duplication. From the collinear data analyses, we detected 18,619 (EXPA-EXPA), 3078 (EXPB-EXPB), 915 (EXLA-EXLA), and 1546 (EXLB-EXLB) pairs of collinear genes (Figure 2, Table S5). The number of collinear *expansin* pairs between two angiosperm species varied widely, from 1 to 194 (*B. napus* versus *Brassica rapa*). The number of collinear *expansin* pairs in the eudicots was larger than that in the basal angiosperms and monocots. Distinct from angiosperms, the *expansin* genes in the chlorophyte algae, streptophyte algae, liverworts, mosses, lycophytes, ferns, and gymnosperms showed no collinearity with each other (Figure 2). In addition, no evidence of collinearity of *expansin* genes was found between the angiosperms and chlorophyte algae, streptophyte algae, liverworts, mosses, lycophytes, ferns, or gymnosperms.

Next, the intraspecies collinear blocks were analyzed (Table S6). We found that 1142 *expansin* genes from 46 species, including *Chara braunii*, *Spirogloea muscicola, P*. *patens*, and 43 angiosperms, were present in 751 collinear blocks, which was significantly lower than the number observed in intergenomic comparisons. Over 98.73% of the collinear *expansin* pairs resulted from angiosperms. However, the collinearity of *expansin* genes was not detected in *A. trichopoda*, one of the two basal angiosperms. Our results suggest that two *expansin* genes (*GBG70289* and *GBG74093*) in *C*. *braunii* might have evolved through WGD or segmental duplication. However, Nishiyama et al. demonstrated that WGD was absent in *C*. *braunii* [35]. Therefore, it is likely that *GBG70289* and *GBG74093* resulted from segmental duplication. Moreover, nine collinear *expansin* pairs in *S. muscicola* could be attributed to a whole genome triplication event [36]. In *P*. *patens*, two WGD events (WGD1 and WGD2) occurred approximately 40–48 and 27–35 million years ago [37], which may have contributed to the increase in *expansin* gene numbers (*Pp3c18_19690V3.2* and *Pp3c21_1890V3.1*, *Pp3c8_13450V3.1* and *Pp3c24_9250V3.1*, *Pp3c1_37980V3.2* and *Pp3c2_3390V3.1*).

To gain further insight into the enlargement of the expansin family, tandem duplication genes (TDGs) were identified (Table S7). A total of 753 *expansin* genes from 50 species were grouped into 256 TDG clusters. In particular, TDG clusters in eight Gramineae plants (an average of ~18 TDG clusters) were larger than those in the other 42 species (an average of ~2.67 TDG clusters). The largest TDG cluster, containing 10 *expansin* genes, was mapped on the 3B chromosome of wheat, likely resulting from unequal crossing-over events [38]. Additionally, we found that tandem duplication primarily contributed to *expansin* enlargement in the chlorophyte algae, streptophyte algae, liverworts, mosses, lycophytes, ferns, and gymnosperms. In contrast, WGD or segmental duplication played more significant roles in the angiosperms. These results suggest that the expansin family has undergone extensive expansion through both WGD/segmental duplication and tandem duplication over evolutionary time.

### 2.3. Sequence Characteristics, Gene Structure, and Intron Distribution Pattern of Expansin Genes in Plants

To explore how the sequence characteristics of *expansin* genes vary from chlorophyte algae to eudicots, we further analyzed 2461 *expansin* genes from 57 studied species. Generally, the gene length, protein length, molecular weight, and isoelectric point (pI) changed substantially (Table S2). For 68.14% of all the *expansin* genes, the gene length ranged from 1000 bp to 2500 bp. Consistent with previously identified expansin proteins, 91.47% of the total expansins had a protein length from 200 to 300 amino acids. The averages of the protein length and molecular weight were highly conserved within the four expansin subfamilies but varied widely between the chlorophyte algae or streptophyte algae and the eight lineages of land plants (Table S8 and Table S9).

Based on exon-intron organization, gene structure is crucial for elucidating the evolutionary processes of gene families. Among the 2461 *expansin* genes, the exon and intron lengths varied significantly, especially the intron lengths, which ranged from 0 to 40,949 bp (Table S2). At the subfamily level, the average changes in the exon and intron lengths were basically conserved, in contrast to the variations observed at the lineage level (Tables S8 and S9). Additionally, a correlation analysis was performed to examine the relationship between gene length and exon or intron length. Exon length had a slight influence on gene length (*r*^2^ = 0.22, *p* < 0.01), whereas intron length had a stronger impact (*r*^2^ = 0.87, *p* < 0.01), implying that changes in gene length are closely related to changes in intron length.

**Figure 3:**
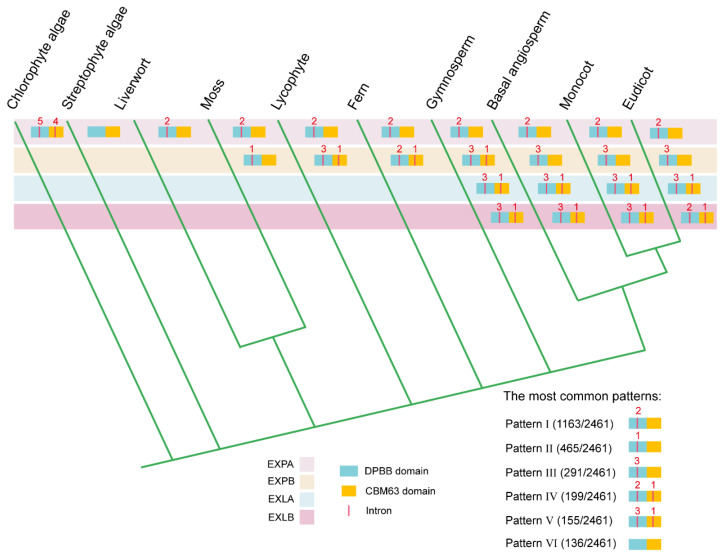
Intron distribution patterns within the DPBB and CBM63 domains across ten lineages. A simplified phylogeny of green plants was drawn according to Leebens-Mack JH et al. and Jia Q et al. [33,39]. Introns in the DPBB and CBM63 domains are indicated by red vertical lines. The red numerals represent the number of introns corresponding to the most common pattern (i.e., the largest percentage) in each subfamily across different lineages.

Subsequently, intron distribution patterns within the DPBB and CBM63 domains were analyzed (Figure 3, Table S10). A total of 2409 (97.89%) *expansin* genes featured the six most common patterns: Pattern I (47.26%), Pattern II (18.89%), Pattern III (11.82%), Pattern IV (8.09%), Pattern V (6.30%), and Pattern VI (5.53%). Pattern I, characterized by two introns in the DPBB domain, was highly conserved in the EXPA from the liverworts to eudicots, indicating that it might have been inherited from the common ancestor of land plants. Pattern II, which features one intron in the DPBB domain, was the second most common pattern in EXPA. EXPB exhibited various distribution patterns during evolution. However, Pattern III, with three introns in the DPBB domain, was the most prevalent in the EXPB from the basal angiosperms to eudicots. In EXLA and EXLB, the distribution patterns were generally conserved, featuring three introns in the DPBB domain and one intron in the CBM63 domain (Pattern V), except for the EXLB in the eudicots. *Expansin* genes from the chlorophyte algae possessed at least six and up to eleven introns in the two domains, differing significantly from those in the streptophyte algae and land plants. The intron distribution patterns in EXPA, EXPB, and EXLA/EXLB were highly conserved in the land plants, angiosperms, and seed plants, respectively. The change in intron number in the DPBB and CBM63 domains suggested that intron gain and loss events were common in the expansin family, from unicellular algae to angiosperms.

### 2.4. Evolutionary Conservation of the Expansin Family

**Figure 4:**
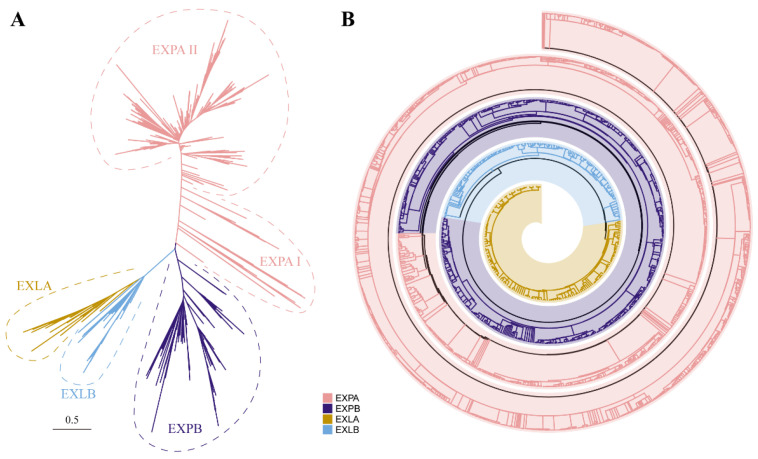
Phylogenetic analysis of expansins in green plants. (**A**) Phylogenetic tree topology of 2461 expansins across 57 species. Different clades within the EXPA subfamily are denoted by Roman numerals. The tree was constructed using IQ-TREE with the best-fitting model (Q.pfam + R9) and visualized using iTOL v5. The scale bar indicates an evolutionary distance of 0.5 nucleotides per position in the sequence. (**B**) Spiral diagram of the phylogenetic tree of 2461 expansins across 57 species. Each subfamily is labeled with a specific color. The tree was constructed using IQ-TREE with the best-fitting model (Q.pfam + R9) and visualized using the R package *spiralize* (v1.1.0). The phylogenetic tree, including all bootstrap values, is presented in Figure S2.

To determine the phylogenetic relationship of plant expansins, 2461 expansin proteins were used to construct an unrooted phylogenetic tree using the maximum likelihood (ML) approach in the IQ-TREE tool. The phylogenetic analysis classified these proteins into five clades: EXPA I, EXPA II, EXPB, EXLA, and EXLB (Figure 4A). Among these, EXPA I was an ancestral clade and clearly distinct from the other clades. Clade EXPA I, the earliest diverging clade, included not only chlorophyte algae (*V*. *carteri* and *C*. *subellipsoidea*) but also streptophyte algae (*Klebsormidium nitens*, *C. braunii*, *S*. *muscicola*, *Mesotaenium endlicherianum*, and *Penium margaritaceum*). These results indicate that all the *expansin* genes from the land plants were derived from the common ancestors of both chlorophyte algae and streptophyte algae.

In parallel, the spiral phylogenetic tree clearly divided all the expansin proteins into four groups: the EXPA group, EXPB group, EXLA group, and EXLB group, demonstrating that expansin proteins from the same subfamily clustered together (Figure 4B and Figure S2). A total of 1610 EXPA members, 557 EXPB members, 139 EXLA members, and 155 EXLB members were assigned to their respective groups (Table S2). The phylogenetic classification was completely consistent with the genome-wide identification of the four subfamilies, revealing that the functional domains from different subfamilies are highly conserved.

In addition, the conservation of two domains (DPBB domain and CBM63 domain) was analyzed by sequence alignment (Figure S3). Among the four subfamilies, the DPBB domain exhibited the highest degree of conservation, possessing a GACG motif and a CGAC motif interspaced by ~30 amino acids. A highly conserved HDF motif was present in the EXPA and EXPB subfamilies, while the TDF motif was observed in the EXLA and EXLB subfamilies. Similar to previous results [15], the CDRC motif in the N-terminus was found only in the EXLA subfamily. We speculate that these conserved motifs are likely involved in the biological functions of the expansin family.

### 2.5. Strong Negative Selection for Expansin Genes

**Figure 5:**
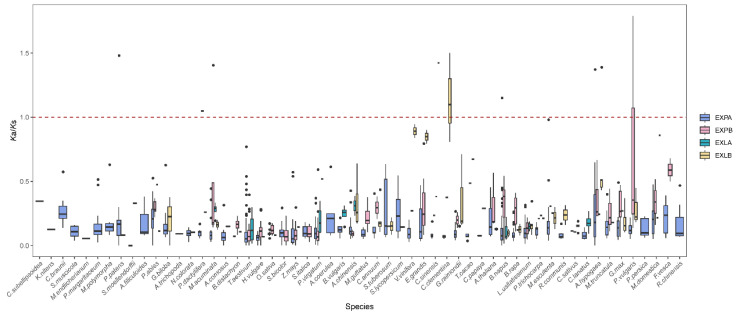
Distribution of *K*a/*K*s values for *expansin* genes. A *K*a/*K*s analysis was performed on paralogous gene pairs across 56 species. The horizontal dashed line indicates the threshold for distinguishing between negative and positive selection in *expansin* genes.

The ratio of nonsynonymous substitution rate (*K*a) to synonymous substitution rate (*K*s) is commonly used to study the type of Darwinian selection. To elucidate the potential selective pressure on *expansin* genes in plants, we calculated the *K*a/*K*s ratio for 1130 paralogous gene pairs from 56 species (except for *V*. *carteri*). The *K*a/*K*s values for these gene pairs ranged from 0.001 to 1.78945 (Figure 5, Table S11). Notably, the paralogous gene pair *Phvul.010G070900*-*Phvul.010G071000* from *Phaseolus vulgaris* had the highest *K*a/*K*s value. The overwhelming majority of gene pairs (99.12%) had *K*a/*K*s values lower than 1, with 524 pairs showing values less than 0.1. These results indicate that most paralogs underwent negative selection with limited functional divergence. Only ten gene pairs (0.88%) from eight species (one in mosses, two in monocots, and five in eudicots) displayed *K*a/*K*s values greater than 1, suggesting that these genes experienced positive selection. Overall, *expansin* genes have been strongly conserved during evolution, reflecting their crucial role in cell wall loosening in plant lineages.

### 2.6. Expression Pattern Analysis of the Expansin Family from Green Algae to Eudicots

**Figure 6:**
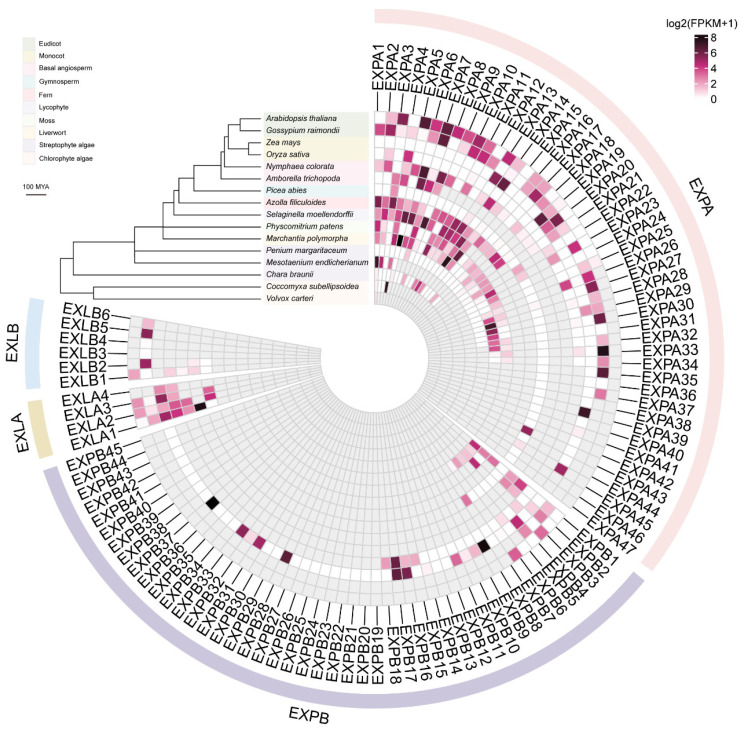
Expression analysis of the expansin family. FPKM values of 506 *expansin* genes from four different subfamilies across 16 species were analyzed. *Expansin* genes in each species were renamed based on the order in their genomic coordinates (e.g., *EXPA1*, *EXPA2*, etc.). Gray quadrilaterals indicate no genes in the corresponding species. The phylogenetic tree of the 16 species was constructed using TimeTree 5 [34]. The scale bar indicates divergence time (million years ago, MYA).

Changes in gene expression patterns are associated with the evolution of gene families. To understand the evolution of the expansin family at the expression level, we analyzed transcriptome data from 16 species across ten lineages (Table S12). Of the 506 *expansin* genes identified in these species, 198 (39.13%) were expressed (FPKM ≥ 1), 97 (19.17%) were expressed at extremely weak levels (0 < FPKM < 1), and 211 (41.70%) were not expressed (FPKM = 0). At the species level, only one *expansin* gene in *V*. *carteri*, *Vocar.0022s0205.1* (*EXPA1*), was found to have extremely weak expression (FPKM = 0.7031) (Figure 6, Table S13). In comparison, *C*. *subellipsoidea* had three additional *expansin* genes. All four *expansin* genes in *C*. *subellipsoidea* showed a large change in their expression levels, with FPKM values ranging from 0 to 169.8159. When analyzing the expression levels of *expansin* genes from the remaining 14 species, we observed a similar trend to that in *C. subellipsoidea*, except for *P. margaritaceum*. This trend was characterized by one or several *expansin* genes with very high expression levels. These genes were defined as specifically or preferentially expressed *expansin* genes that played key roles in development and function. Together with the expansion of the expansin family, we propose that changes in the expression levels of *expansin* genes were accompanied by functional conservation and diversification during species evolution.

### 2.7. qRT-PCR Validation of Expansin Genes in Various Maize Tissues

**Figure 7:**
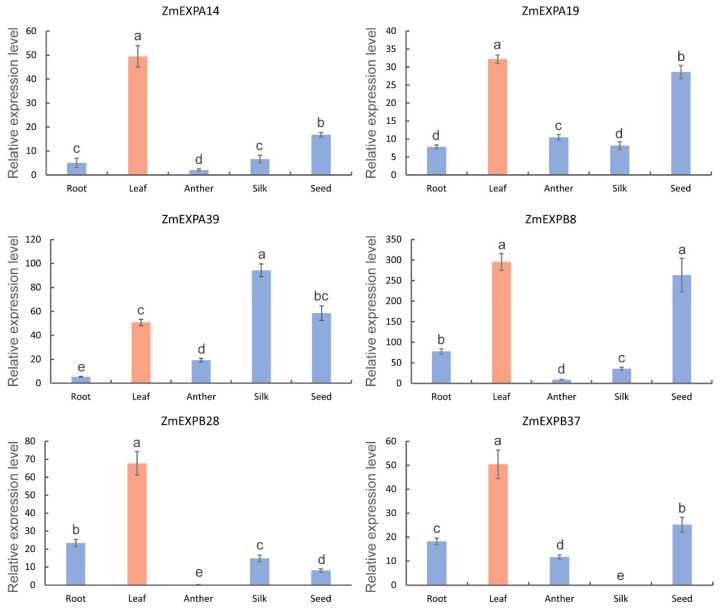
qRT-PCR analysis of the expression patterns of six *expansin* genes in five maize tissues. All expression levels were normalized to *ZmEF1α*. The rose-red and blue bars represent the relative expression levels of *expansin* genes in leaves and the other four tissues, respectively. Error bars indicate the standard deviation of three biological replicates. Different letters represent statistically significant differences at *p* < 0.05 based on ANOVA (Duncan’s multiple comparison test).

To further validate the expression patterns of *expansin* genes in leaves, six genes (*ZmEXPA14*, *ZmEXPA19*, *ZmEXPA39*, *ZmEXPB8*, *ZmEXPB28*, and *ZmEXPB37*) with high expression levels in maize leaves were selected based on the transcriptome data. We employed qRT-PCR to analyze the expression characteristics of these *expansin* genes across five tissues: root, leaf, anther, silk, and seed, in the maize inbred B73. The results demonstrate that the expression patterns of these genes were consistent with the transcriptome data, showing high expression levels in maize leaves (Figure 7). Additionally, various transcript levels were observed for the six genes across the different tissues. For example, five genes (*ZmEXPA14*, *ZmEXPA19*, *ZmEXPA39*, *ZmEXPB8*, and *ZmEXPB37*) showed high expression in the seeds, especially *ZmEXPB8*, suggesting that *expansin* genes may play a role in seed development. *ZmEXPA39* also exhibited the highest expression in the silk. However, *ZmEXPB28* and *ZmEXPB37* were absent in the anther and silk, respectively. These findings enhance our understanding of the functional conservation and diversification of *expansin* genes during evolution.

## 3. Discussion

Expansin, a plant-specific protein that facilitates cell wall loosening, has critical functions in various biological processes by promoting cell elongation and expansion, as well as regulating development. Despite the recognized importance of expansins, the origin, evolution, and diversification of the expansin family in plants have yet to be systematically analyzed. In this study, we identified a total of 2461 expansin proteins from 64 species across eleven lineages at the genome-wide level. By investigating the collinearity and phylogenetic relationships, sequence characteristics, and gene expression patterns, our aim was to deepen the understanding of their evolutionary history and functional diversification in green plants.

The genome-wide gene identification suggested that expansins first appeared in Chlorophyta, followed by a significant increase in the size of the expansin family in Streptophyta (Figure 1, Table S2). Unexpectedly, homologous genes to *Arabidopsis* EXPA were found only in two chlorophyte algae (*V. carteri* and *C. subellipsoidea*) and five streptophyte algae (*K. nitens*, *C. braunii*, *S. muscicola*, *M. endlicherianum*, and *P. margaritaceum*) but not in the other three chlorophyte algae (*O. lucimarinus*, *M. pusilla*, and *C. reinhardtii*) and two streptophyte algae (*M. viride* and *C. atmophyticus*). Based on the phylogenetic relationship of the expansin family (Figure 4A), we speculate that Clade EXPA I might be the common ancestor of land plant expansins. Additionally, it is highly probable that these two chlorophyte algae and five streptophyte algae employed EXPA for cell wall loosening, similar to land plants. Therefore, we presume that EXPA in chlorophyte algae and streptophyte algae laid the foundation for the cell wall loosening function in land plants.

In green plants, horizontal gene transfer (HGT) is a crucial way to acquire novel genes from bacteria or fungi [40]. In recent years, over 200 horizontally acquired genes or gene families have been identified across various plant taxa [41]. For example, TAL-type transaldolase (associated with vascular development), peroxiredoxin (involved in stress response), and several cell wall-related genes, including glycoside hydrolase subfamily GH5_11 (related to cellulose degradation), *β*-glucosidase (involved in cellulose degradation), and glycoside hydrolase family 43 *β*-xylosidase (associated with xylan degradation) [41]. The DPBB domain and the CBM63 domain are two canonical expansin domains [13]. We found that the DPBB domain, but not the CBM63 domain, was present in one red alga (*C. crispus*) and three chlorophyte algae (*O. lucimarinus*, *M. pusilla*, and *C. reinhardtii*), all of which lacked expansin proteins (Figure S1, Table S3). This finding implies that the DPBB domain may have originated in red algae or chlorophyte algae. However, it is also possible that the CBM63 domain in chlorophyte algae resulted from an HGT event from an unknown bacteria or fungi.

Gene duplication contributes to increased genome complexity during evolution [42]. WGD, segmental duplication, and tandem duplication are common duplication events that drive gene family expansion. Many studies suggest that both recent and ancient polyploidization events have repeatedly occurred across all major land plant lineages, with WGD being particularly prevalent in angiosperms [43]. In this study, tandem duplication was identified as the major driving force behind the enlargement of the expansin family in chlorophyte algae, streptophyte algae, liverworts, mosses, lycophytes, ferns, and gymnosperms (Table S7). In contrast, in angiosperms, the expansin family expanded mainly through WGD/segmental duplication (Figure 2, Tables S5 and S6), indicating that WGD/segmental duplication had important consequences for gene family expansion during angiosperm evolution, aligning with previous findings [43].

Gene loss is important in green plant evolution. It is not only associated with gene redundancy but also with phenotypic diversity [44]. With the sequencing of numerous plant genomes, gene loss has been observed in various plant species, such as *Cuscuta australis*, three *Brassica* species [45,46]. By far, the role of the EXLB subfamily remains poorly understood in green plants. It has been reported that the entire EXLB subfamily was lost in three aquatic plant species, likely as an adaptation to their aquatic environment [47]. Among the eleven monocots studied, the complete absence of the EXLB subfamily was found only in four Gramineae crops: *T. aestivum*, *H. vulgare*, *S. bicolor*, and *Z. mays* (Figure 1). One reason for this loss may be the development of specialized physiological traits that are better suited to their living environment. Another possibility is that EXLB may have become dispensable for the growth and development of the four Gramineae crops. The underlying cause of EXLB loss, however, necessitates further exploration.

Gene duplication and loss can drive divergence in gene expression and promote functional diversification. Among the 16 species studied, with the exceptions of *V. carteri* and *M. endlicherianum*, expansin members exhibited different expression patterns (Figure 6). Expansin genes with high expression levels were considered to play important roles in regulating growth and development. Conversely, the 211 non-expressed *expansin* genes (FPKM = 0) were likely pseudogenes or may lack functionality in cell wall loosening. However, it is also possible that these genes could be expressed under specific conditions. They are thought to serve as a reservoir of genetic material, offering organisms adaptability to changing conditions and environmental pressures [48]. Additionally, a qRT-PCR analysis of various maize tissues confirmed the functional conservation and diversification of *expansin* genes during evolution (Figure 7).

In summary, our study reveals the early origin and evolutional divergence of the expansin family, while also uncovering the patterns of gene duplication and expression among plant expansins. This comprehensive analysis enhances our understanding of their functions from an evolutionary perspective.

## 4. Materials and Methods

### 4.1. Genome-Wide Identification of Expansin Genes among 64 Species

The genome files of 64 species were downloaded from various databases between 2022 and 2023 (Table S1). To identify *expansin* genes across these species, 35 expansin proteins from *Arabidopsis thaliana* (excluding the pseudogene *AtEXPA19*) were collected from TAIR (https://www.arabidopsis.org/index.jsp, accessed on 7 January 2022) and used as queries to search for homologous genes in the other 63 species via BLASP (v 2.10.1+) analysis. The parameters for BLASP were set to “sequence coverage >30%, identity >30%, and e-value < 1 × 10^−3^”, while other parameters were maintained at the default values [49]. Subsequently, hidden Markov model profiles for PF03330 (DPBB domain) and PF01357 (CBM63 domain) were obtained from the Pfam database (http://pfam.xfam.org/, accessed on 13 January 2022). HMMER 3.3.1 was used to detect protein domains with an e-value < 1 × 10^−5^ [50]. The protein domains were further searched using the NCBI CDD in default mode (https://www.ncbi.nlm.nih.gov/cdd/, accessed on 23 January 2022 and 8 July 2023) [51]. Only protein sequences containing both the DPBB and CBM63 domains were retained. Due to alternative splicing, some *expansin* genes had multiple transcripts. Thus, the proteins with the longest lengths were chosen, except for six proteins with appropriate lengths (Table S14). Moreover, eleven proteins with misannotated flanking regions were reannotated using the gene prediction software FGENESH (v2.6) and assigned new names (Table S15) [52]. Finally, 2461 *expansin* genes were identified for further analysis (Table S2). To visualize changes in the gene copy number across the 64 species, a species tree with divergence times was constructed using TimeTree 5 (http://www.timetree.org/, accessed on 10 July 2023) [34] and visualized using Evolview v3 [53].

### 4.2. Phylogenetic Analysis

A total of 2461 expansin protein sequences were aligned using the Muscle5 (v5.1) program with the parameters “muscle -align fasta.file -output aligned_fasta.file -perturb 0 -perm none -consiters 2 -refineiters 100” [54]. The phylogenetic tree was constructed using the IQ-TREE (v1.6.12) software with the best-fitting model (Q.pfam + R9) [55]. The parameters for IQ-TREE were set to “-m MFP -bb 1000 -bnni -nt AUTO -cmax 15 -redo”. The bootstrap value was 1000. The topology of the phylogenetic tree was visualized using iTOL v5 [56]. The R package *spiralize* (v1.1.0) was used to visualize the phylogenetic tree, displaying 2461 genes on the spiral [57].

### 4.3. Gene Structure and Sequence Conservation Analysis

The R package *rtracklayer* (v1.48.0) was used to obtain general feature format version 3 (GFF3) files from 57 species genomes [58]. The exon-intron structure information of the *expansin* genes was then extracted from these GFF3 files. The gene length, protein length, exon length (total exon length per gene), exon number, intron length (total intron length per gene), and intron number were calculated. The molecular weight and pI of the putative expansin proteins were calculated using the online tool, IPC 1.0 (http://isoelectric.org/, accessed on 29 January 2022 and 11 July 2023) [59]. Furthermore, the intron distribution within the DPBB and CBM63 domains was investigated as described by Xu et al. [60].

To investigate the conservation of the two domains, multiple sequence alignment was performed using Muscle5 (v5.1) [54]. Signal peptides and poorly conserved amino acids in the predicted expansin proteins were manually removed. The conserved amino acids within the protein sequences were visualized using the R package *ggseqlogo* (v0.1) [61].

### 4.4. Gene Duplication and Synteny Analysis

The chromosomal positions of the *expansin* genes were identified using GFF3 files. Tandem duplication genes were defined as genes separated by no more than one intervening gene on the same chromosome [49]. Following Maher et al. [62], 20 protein-coding genes located upstream and downstream of each *expansin* gene were extracted to determine large-scale duplication events. The collinearity among genomic regions containing 41 protein-coding genes was analyzed using MCScanX with default parameters [63].

### 4.5. Calculation of Ka/Ks Values

A multiple sequence alignment of 2461 expansin proteins was prepared for *K*a/*K*s estimation using Muscle5 (v5.1) and ParaAT (v2.0) [54,64]. The *K*a/*K*s ratio for each paralogous gene pair was then calculated with KaKs_Calculator 3.0 [65]. To avoid the risk of saturation effects, gene pairs with *K*s values greater than 2.0 were discarded [66]. A *K*a/*K*s ratio greater than one indicates a positive or diversifying selection, while a ratio lower than one represents a negative or purifying selection. *K*a/*K*s = 1 indicates neutral evolution.

### 4.6. Transcriptome Data Analysis

Transcriptome data from sixteen species across ten lineages were downloaded from the NCBI SRA database (https://www.ncbi.nlm.nih.gov/sra/?term=, accessed on 5 March 2022 and 13 July 2023) (Table S12). For *V. carteri*, *C. subellipsoidea*, *M. endlicherianum*, and *P. margaritaceum*, whole plant (unicellular organism) was selected. In *C. braunii* and *Azolla filiculoides*, the thallus was chosen. For *P. patens*, *Selaginella moellendorffii*, *A*. *filiculoides* and *P. abies*, the protonema, vascular leaf, sporophytes, and needles were selected, respectively. In *A. trichopoda*, *Nymphaea colorata*, *O. sativa*, *Z. mays*, *A. thaliana*, and *Gossypium raimondii*, the leaf was selected. Quality control of the raw reads was performed using Trimmomatic-0.39 [67]. The clean reads were further aligned to the genomes using HISAT2 (Version 2.2.0) [68]. The gene expression levels were estimated using StringTie2 (Version 2.1.4) and normalized by FPKM [69]. A heatmap was drawn using shinyCircos-V2.0 based on the gene expression level data (FPKM) [70]. A species tree with divergence times was constructed using TimeTree 5 (http://www.timetree.org/, accessed on 18 July 2023) [34].

### 4.7. RNA Isolation and qRT-PCR Analysis

In this study, maize inbred B73 was used to analyze the expression patterns of *expansin* genes across five tissues: primary roots (8 days after sowing in a growth chamber), young leaves (18 days after sowing in a growth chamber), anthers (field-grown maize), silks (field-grown maize), and seeds (10 days after pollination in the field). Following the user guide, TRIzol^TM^ Reagent (Catalog Number 15596026, Invitrogen, Carlsbad, CA, USA) was used to extract the total RNA from the five maize tissues. Subsequently, cDNA was synthesized from 1 μg of total RNA per sample using SuperScript™ IV (Catalog Number 18090010, Invitrogen, Carlsbad, CA, USA). qRT-PCR was performed on an ABI StepOne Plus Real Time PCR System (Applied Biosystems^®^ Inc., Foster City, CA, USA) using MonAmp^TM^ SYBR Green qPCR Mix (Catalog Number MQ10201S, Monad, Wuhan, China). *ZmEF1α* served as an internal control, and the primers used for qRT-PCR are listed in Table S16.

## 5. Conclusions

Expansin is a pivotal factor in loosening the cell wall in green plants. In this study, we examined the origin, evolution, and diversification of the plant expansin family at the genome level. Our findings indicate that plant *expansin* genes originated from chlorophyte algae, with subsequent expansion of the gene family occurring through WGD/segmental duplication and tandem duplication events. A gene structure analysis revealed relatively conserved intron distribution patterns across different subfamilies. A phylogenetic analysis supported the origin of expansins and the conservation of four subfamilies. The expression patterns of *expansin* genes across 16 species, along with qRT-PCR analysis in maize, indicated that the functional conservation and diversification of *expansin* genes were accompanied by gene expansion during evolution. This study elucidates the molecular evolution of plant expansins from a bioinformatics perspective, enhancing our understanding of their biological functions and cell wall evolution.

## Data Availability

Data are contained within the article or Supplementary Materials.

## Supplementary Materials

The following supporting information can be downloaded at: https://www.mdpi.com/article/10.3390/ijms252111814/s1.
