# Supplementary material for: Origin, Evolution, and Diversification of the Expansin Family in Plants
Source: Int J Mol Sci. 2024 Nov 3;25(21):11814. doi: 10.3390/ijms252111814 (PMC11547041; doi:10.3390/ijms252111814)
Supplement: Supplementary file 1 [file ijms-25-11814-s001.zip › Supplementary Figures.pdf]

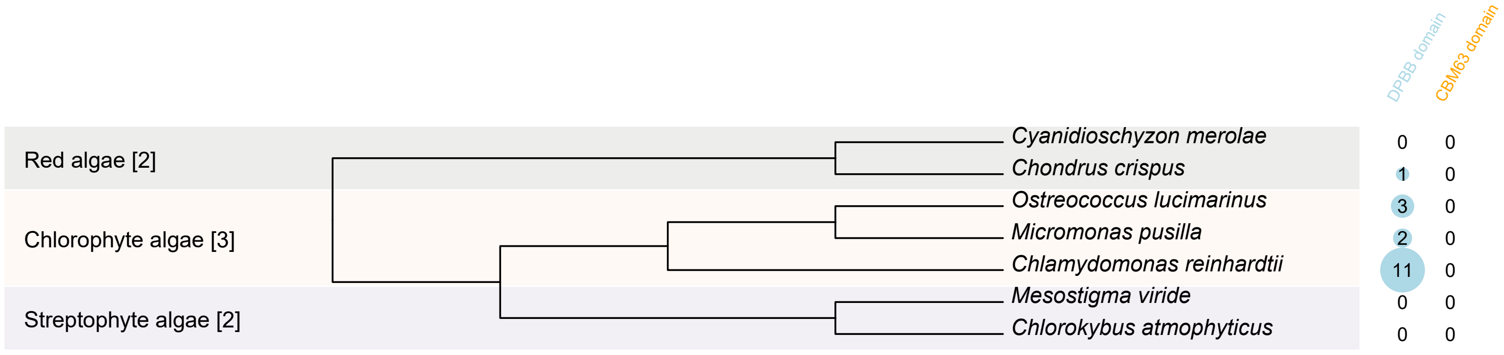

Figure S1 Occurrences of the DPBB and CMB63 domains in two red algae, three green algae, and two streptophyte algae.

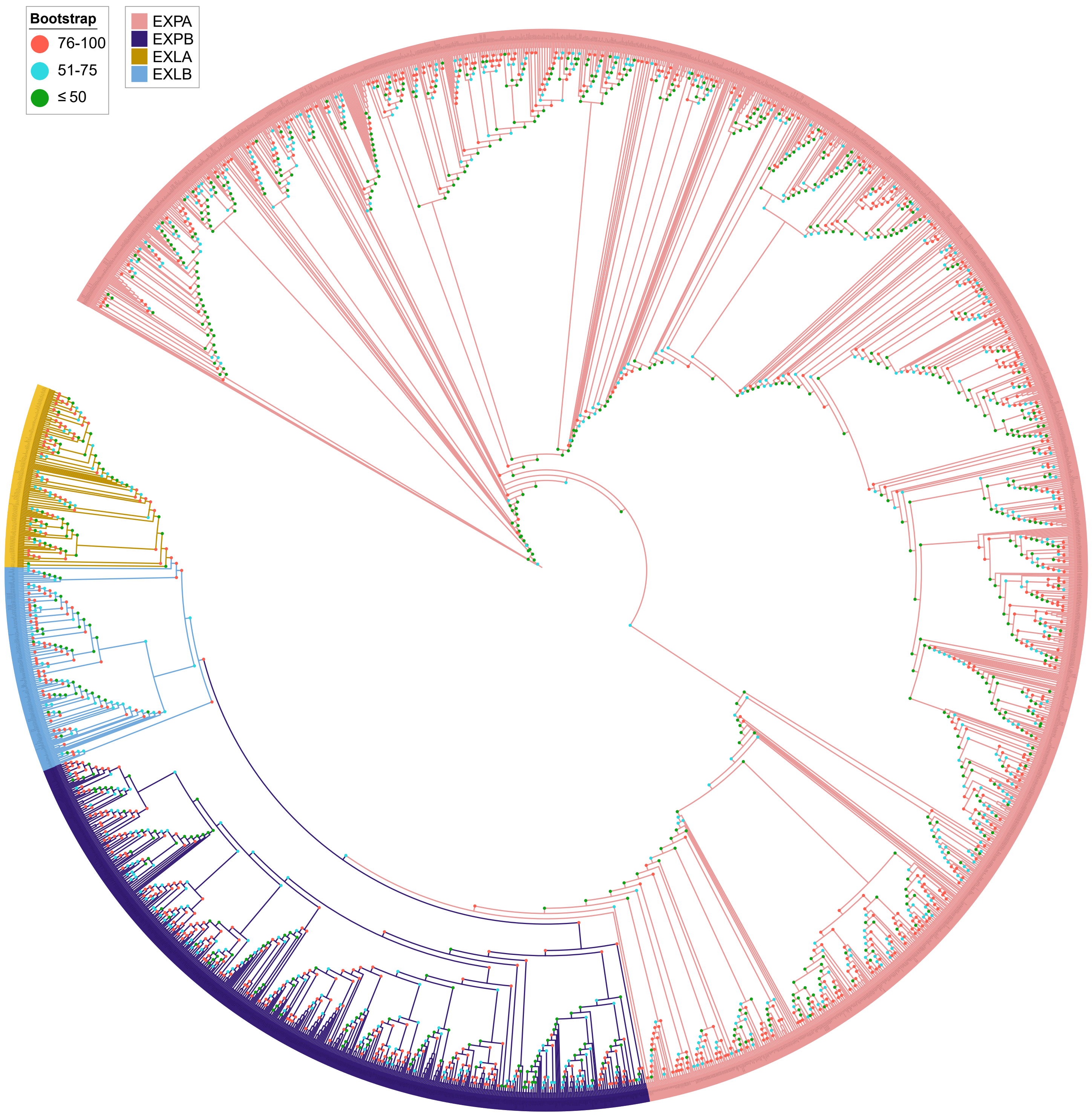

Figure S2 Phylogenetic relationship of the expansin family across 57 species.
A maximum-likelihood phylogram of expansin protein sequences was constructed with 1,000 bootstrap replicates. Red, blue, and green circles indicate node support values of 76-100, 51-75, and ≤50, respectively. The tree was visualized using iTOL v5.

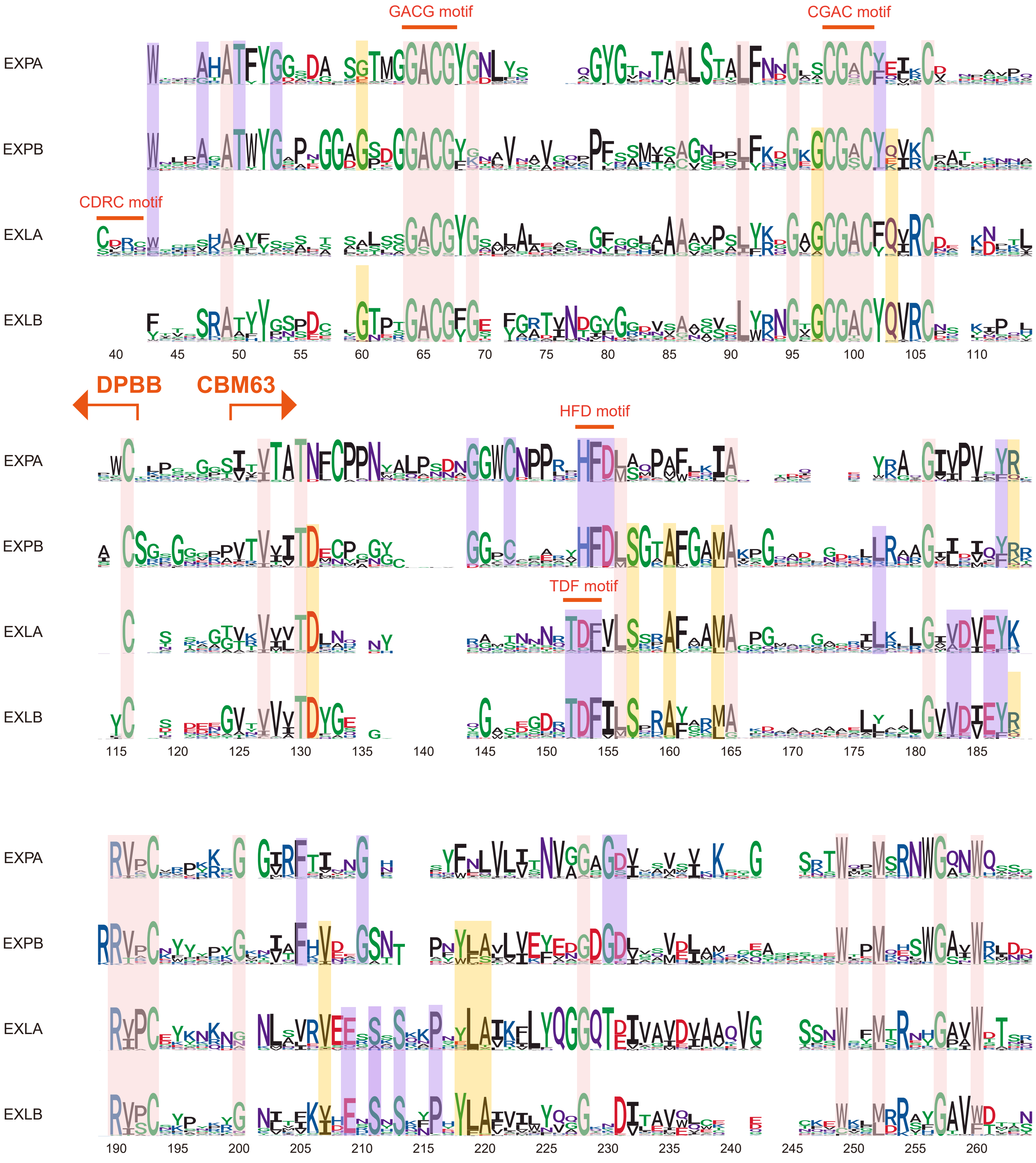

Figure S3 Conserved amino acid sequences in the four expansin subfamilies.
The height of the amino acid symbols at each position represents the degree of sequence conservation. The numbers at the bottom of the sequences indicate the amino acid positions. Conserved amino acid residues among different subfamilies are shaded in different colors (purple: two subfamilies; yellow: three subfamilies; pink: four subfamilies). The orange arrows represent the boundary between the DPBB domain and the CBM63 domain.
